# Patient Blood Management Adoption and Transfusion-Threshold Concordance Among Romanian Anesthesiologists: National Survey Analysis

**DOI:** 10.2196/106731

**Published:** 2026-10-16

**Authors:** Mihai Gabriel Stefan, Daniela Filipescu, Dan Corneci, Cornelia Predoi, Silvius Negoita, Dorel Sandesc, Liana Valeanu, Serban Ion Bubenek Turconi, Stefan Andrei, Dana Tomescu

**Affiliations:** 1 Discipline of Anaesthesiology and Intensive Care Medicine, Carol Davila University of Medicine and Pharmacy, Bucharest, București, Romania; 2 Department of Anaesthesiology and Intensive Care Medicine, Emergency Institute for Cardiovascular Diseases "Prof. C.C. Iliescu", Sos Fundeni, 258, Bucharest, București, 022322, Romania, 40 0213021110; 3 Department of Anaesthesiology and Intensive Care, Spitalul Universitar de Urgenţă Militar Central Dr. Carol Davila, Bucharest, București, Romania; 4 Department of Anaesthesiology and Intensive Care, Spitalul Universitar de Urgență Elias, Bucharest, București, Romania; 5 Department of Anaesthesiology and Intensive Care, Victor Babeș University of Medicine and Pharmacy Timișoara, Timisoara, Timiș County, Romania; 6 Department of Anaesthesiology and Intensive Care, Spitalul Clinic Judeţean de Urgenţă, Timisoara, Timiș County, Romania; 7 Department of Anesthesia and Perioperative Care, University of California, San Francisco, San Francisco, United States; 8 Department of Anaesthesiology and Intensive Care, Hôpital Bichat-Claude-Bernard, Paris, Île-de-France, France; 9 Group of Data Modeling, Computational Biology, and Predictive Medicine, Applied Mathematics, Institut de Biologie de l'École Normale Supérieure, Paris, Île-de-France, France; 10 Department of Anaesthesiology and Intensive Care, Institutul Clinic Fundeni, Bucharest, București, Romania

**Keywords:** patient blood management, transfusion, anemia, hemoglobin threshold, anesthesiology, survey, implementation, guideline adherence, Romania

## Abstract

**Background:**

Romania issued national patient blood management (PBM) guidelines in 2018. A 2023 national survey described the overall uptake of PBM among anesthesiologists but did not examine which clinicians and institutions adopted PBM, nor whether stated transfusion thresholds aligned with a study-defined restrictive reference.

**Objective:**

This study aimed to identify determinants of PBM adoption and to quantify transfusion-trigger concordance, using a planned, exploratory, cross-sectional secondary analysis of the 2023 survey.

**Methods:**

We analyzed 511 responses to the 38-item Romanian Society of Anaesthesia and Intensive Care (SRATI) PBM survey. Three adoption outcomes (applying PBM, a formal hospital PBM group, and routine preoperative anemia treatment) were modeled by multivariable logistic regression against physician grade, hospital category, and local PBM educational events. Eight standardized clinical vignettes (age×cardiac disease×bleeding state) were scored against a restrictive hemoglobin reference (7 g/dL general; 8 g/dL with cardiac disease). The reported timing of the preanesthetic consultation (4 frequency items) was additionally examined as a determinant of preoperative anemia treatment. This was a planned, exploratory secondary analysis; the robustness of the main findings was examined in sensitivity analyses that accounted for survey attrition and for alternative transfusion-threshold scoring rules.

**Results:**

Overall, 73.7% (373/506) reported applying PBM, 33.1% (158/477) reported a formal PBM group, and 33.5% (117/349) reported routine preoperative anemia treatment. Local PBM educational events were the strongest adjusted correlate of reported adoption (applying PBM: adjusted odds ratio [aOR] 9.78, 95% CI 4.33‐22.07; *P*<.001; formal group: aOR 7.72, 95% CI 4.30‐13.88; *P*<.001). Earlier preanesthetic consultation was associated with routine preoperative anemia treatment (aOR 1.79 per frequency level for consultation more than 7 days before surgery, 95% CI 1.36‐2.35; *P*<.001). Transfusion-trigger concordance was lower for older than younger patients in otherwise identical scenarios (eg, active bleeding without cardiac disease: 195/345, 56.5% at ≥65 years vs 288/347, 83% at <65 years), and 56.5% (196/347) endorsed a liberal trigger (hemoglobin ≥8 g/dL) in at least one noncardiac scenario. Self-reported PBM application was not associated with a restrictive transfusion pattern (aOR 1.17, 95% CI 0.69‐1.98; *P*=.56). Findings were unchanged across scoring rules, and reported PBM application was similar among respondents who did and did not complete the demographic block (246/329, 74.8% vs 127/177, 71.8%; *P*=.53).

**Conclusions:**

In this exploratory cross-sectional analysis, reported PBM adoption was associated more strongly with local educational activity than with hospital category or physician seniority. Self-reported PBM application was not associated with restrictive stated thresholds in hypothetical scenarios, and respondents selected more liberal thresholds for older patients. Standardized multidisciplinary pathways, earlier anemia screening shared with the surgical team, and education focused on transfusion decisions may help address these gaps. Prospective studies using observed process measures, transfusion practice, and clinical outcomes are needed.

## Introduction

Patient blood management (PBM) is a patient-centered, evidence-based approach to improve outcomes and the safety of patient care. It rests on 3 pillars: detecting and treating anemia, minimizing blood loss and bleeding, and harnessing physiological tolerance of anemia through judicious, restrictive transfusion [1,2].

Multimodal PBM programs are associated with reduced transfusion and improved outcomes [3,4]. Moreover, large randomized trials and meta-analyses have shown that restrictive red blood cell transfusion strategies are, for most patient groups, as safe as liberal strategies while reducing exposure to allogeneic blood [5-10]. International bodies, including the World Health Organization, have called for the urgent and systematic implementation of PBM and issued a comprehensive guidance document on the subject [11]. Romania issued national PBM recommendations, endorsed by the Romanian Society of Anaesthesia and Intensive Care (SRATI) and the Ministry of Health in 2018, addressing preoperative anemia, perioperative bleeding, and appropriate transfusion [12].

In 2023, 5 years after those recommendations were issued, SRATI conducted a national survey among anesthesiologists to assess PBM implementation. The primary report of that survey described overall uptake, showing, for example, that roughly three-quarters of respondents applied some PBM measures but only a third worked in a hospital with a formal PBM group or routinely treated preoperative anemia [13]. That analysis was, by design, descriptive: it reported the marginal frequency of each response without examining who adopted PBM or whether the transfusion thresholds clinicians endorsed were actually consistent with the restrictive strategy the guidelines recommend.

Two questions therefore remain unanswered and are clinically and organizationally important. First, which clinician- and hospital-level factors are associated with PBM adoption—information needed to target implementation efforts. Second, how closely do stated transfusion triggers align with a study-defined restrictive reference, and does reported PBM application correspond to restrictive stated thresholds? In this planned secondary analysis of the 2023 survey, we addressed both questions, modeling determinants of 3 adoption outcomes and scoring concordance across 8 standardized clinical vignettes.

## Methods

### Study Design and Data Source

This was a planned, exploratory secondary analysis of the 2023 SRATI national PBM survey [13]. The instrument comprised 38 items (37 closed-response items and 1 open-ended item) covering PBM awareness and application, institutional structures, anemia diagnosis and treatment, bleeding management, transfusion practice, perceived barriers, and respondent and hospital characteristics. The SRATI PBM working group developed the survey and tested its usability and technical functionality before fielding. It was administered using SurveyMonkey, with one question displayed per screen. Invitations were distributed by email to approximately 2000 members through the SRATI database, with additional announcements at society events and in the society newsletter. Respondents formed a self-selected convenience sample of anesthesiologists practicing in Romania. Data were collected between March 9 and September 9, 2023; 512 respondents opened the survey, and the analytical dataset contained 511 records. No new data were collected for this analysis. Reporting followed CHERRIES (Checklist for Reporting Results of Internet E-Surveys) [14]; the completed checklist is provided as a separate supplementary file for editorial review. Throughout, we distinguish reported PBM application (individual self-report), formal institutional PBM structures (reported presence of a hospital PBM group), and transfusion-threshold concordance (agreement of stated triggers with the study-defined restrictive reference).

### Ethical Considerations

The parent survey was approved by the SRATI research ethics board (approval 5/3.03.2023). The invitation and introductory page explained the study purpose, investigator, expected completion time, and the planned use and storage of data. Participation was voluntary, questionnaire completion constituted informed consent, and no incentives or compensation were provided. No names or email addresses were collected with survey responses. Although the survey platform recorded IP addresses, these were not used in the analysis, and the analytical dataset contained no direct identifiers. The secondary analysis used the same deidentified dataset covered by the parent approval; no additional consent was required.

### Outcomes and Variables

Three binary adoption outcomes were defined: (1) applying PBM in daily practice (yes vs no), (2) presence of a formal hospital PBM group (yes vs no or uncertain), and (3) routine preoperative anemia treatment in the respondent’s hospital (usually or always vs sometimes, rarely, or never; the item asked whether preoperative anemia treatment other than transfusion was practiced in the respondent’s hospital). Adoption of PBM was self-reported with the single item “Aplicați PBM în practica dumneavoastră curentă?” (“Do you apply PBM in your current practice?”; yes or no), which did not define the individual PBM pillars; all items used in this analysis are reproduced in Romanian and English in Multimedia Appendix 1. Candidate predictors were physician grade (senior consultant, specialist, and resident), hospital category (university or institute, county, municipal or town, and other) and whether PBM educational events had been held locally in the previous 3 years. In an exploratory extension, we also examined the reported timing of the preanesthetic consultation. Four items asked how often the consultation was performed more than 7 days before surgery, more than 1 day but less than 7 days before surgery, on the day before surgery, and on the morning of surgery. Each item used a 5-point frequency scale (never, rarely, sometimes, usually, and always), which we scored 0 to 4 and entered as an ordinal predictor.

### Transfusion-Trigger Concordance

Eight survey items presented a balanced 2×2×2 set of clinical vignettes. The factors were patient age (<65 vs ≥65 years), cardiac disease (absent vs present), and active intraoperative bleeding (present vs absent). The older-patient bleeding vignettes specified patients aged 65 years or older, and the older-patient nonbleeding vignettes specified patients older than 65 years; both were analyzed as the ≥65-year stratum. For each vignette, respondents selected the hemoglobin threshold at which they would routinely transfuse (6, 7, 8, or ≥9 g/dL). The bleeding vignettes specified active intraoperative bleeding; the other vignettes described an anemic patient without active bleeding. Cardiac disease was stated explicitly in every scenario. Hemodynamic status, transfusion-related symptoms, frailty, and other comorbidities were not specified. Full wording is provided in Multimedia Appendix 1. Responses were scored against a study-defined restrictive reference consistent with the 2018 Romanian, European, and international recommendations [2,12,15]: a hemoglobin threshold of 7 g/dL for patients without cardiac disease and 8 g/dL for patients with cardiac disease. Each vignette response was scored with 1 of 3 per-vignette rules: the primary rule (concordant if at or below the reference), a strict rule (concordant only if equal to the reference), and a lenient rule (concordant if within 1 g/dL of the reference). The open-ended ≥9 g/dL option was coded as 9 g/dL; it was therefore discordant under the primary and strict rules and concordant under the lenient rule only when the reference is 8 g/dL. Throughout, “concordance” denotes agreement with this study-defined numerical reference and is not a judgment of the clinical appropriateness of an individual transfusion decision. This applies particularly to the active bleeding vignettes, in which hemodynamic status, bleeding rate, symptoms, and anticipated blood loss also govern transfusion in practice but were not specified. At the respondent level, a restrictive pattern was defined as concordance in at least 6 of the 8 vignettes under the rule in use; the primary rule was used for the main analyses and the strict and lenient rules in sensitivity analyses.

### Statistical Analysis

Categorical variables are summarized as counts and percentages using item-specific denominators. Each adoption outcome was modeled by multivariable logistic regression using a fixed covariate set: physician grade, hospital category, and local educational events. Odds ratios (ORs) were reported with 95% CI, and model discrimination was reported as the area under the receiver operating characteristic curve (AUC). The covariate set was fixed in advance rather than selected from the data. Physician grade and hospital category were included because individual seniority and institutional setting are established determinants of guideline uptake, and local educational activity was included because it is the most consistently reported modifiable driver of PBM implementation. No stepwise or significance-based variable selection was performed, and the same covariates were used for all 3 outcomes; models extended with institutional infrastructure variables are reported in Multimedia Appendix 2. Hospital sector (public or private) was not entered as a covariate because it was strongly collinear with hospital category—private practice occurred almost exclusively within the “other” hospital category—which produced unstable estimates. Within-respondent contrasts between paired vignettes were tested with the McNemar test, and predictors of a restrictive transfusion pattern (concordance on at least 6 of the 8 vignettes) were examined by logistic regression. Because item completion declined across the questionnaire, the proportion applying PBM was compared between respondents who did and did not complete the demographic block to assess informative attrition. Two-sided *P*<.05 was considered significant; for the family of 8 vignette comparisons, the Benjamini-Hochberg procedure was applied. Analyses were performed in Python 3 (Python Software Foundation; NumPy, SciPy, and pandas); logistic regression coefficients were estimated by maximum likelihood with Wald 95% CIs, and single-predictor estimates were verified against the corresponding 2×2 OR. In this exploratory extension, each ordinal consultation timing score was added separately to the fixed covariate set, and its adjusted odds ratio per 1-level increase in frequency was estimated for each adoption outcome. Ordinal frequency scores were entered as linear terms on the log-odds scale; this assumption was verified by refitting the models with the score as a categorical variable and comparing fits by likelihood-ratio test. Models used complete cases for the covariate set, and the number of respondents and outcome events in each model was reported with each estimate.

## Results

### Respondents and Attrition

Of 511 analyzable records, 477 to 506 (93.3-99%) respondents answered the core PBM items near the beginning of the questionnaire. Relative to the approximately 2000 members invited, this corresponds to an approximate invitation-to-response proportion of 26%; the exact number of active email addresses and the number who opened the invitation were unavailable. Fewer respondents completed the demographic block at the end of the questionnaire: 331 reported physician grade, and 333 reported hospital category (Figure 1). Earlier items, including educational exposure and availability of anemia diagnostic tests, had higher completion (n=348 and n=350, respectively; Table 1). Completion of the demographic block was defined by provision of physician grade, the least-completed item (n=331). Reported PBM application was similar among respondents who did and did not complete this block (246/329, 74.8% vs 127/177, 71.8%; *P*=.53), and the complete-case sample for the adjusted models was 326 to 328. Respondents represented 41 of Romania’s 42 county-level administrative units. Among 324 respondents reporting location, 99 (30.6%) practiced in Bucharest.

**Figure 1.**
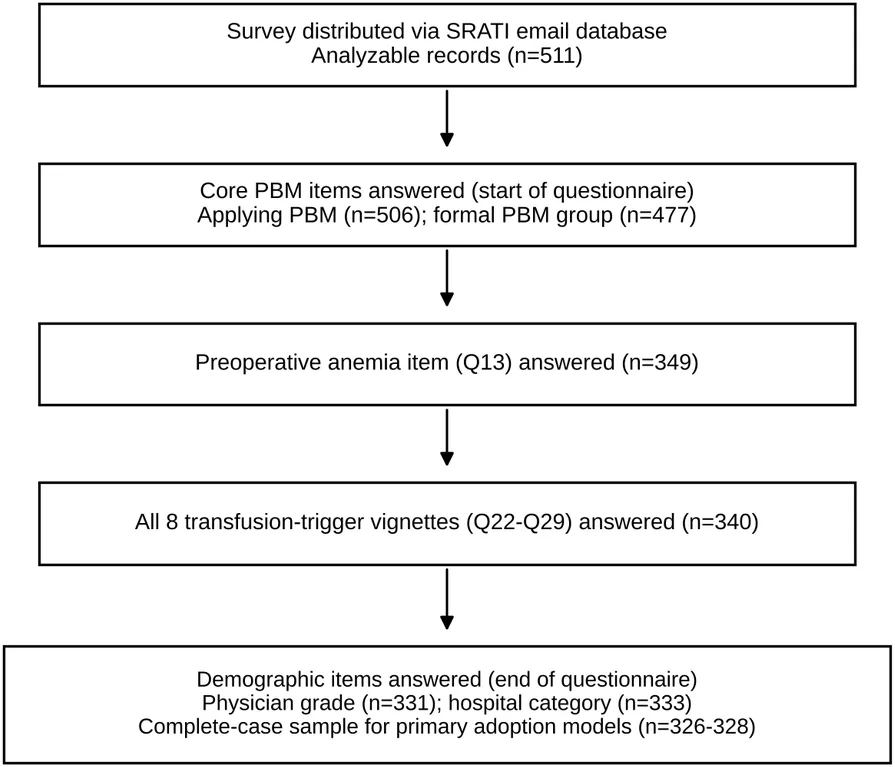
Respondent flow and item completion through the questionnaire. SRATI: Romanian Society of Anaesthesia and Intensive Care.

**Table 1.** Respondent and hospital characteristics^a^.

Characteristics	Respondents
Physician grade (n=331), n (%)^b^	
Senior consultant	175 (52.9)
Specialist	112 (33.8)
Resident	44 (13.3)
Hospital category (n=333), n (%)^b^	
University or institute	130 (39)
County	91 (27.3)
Municipal or town	67 (20.1)
Other	45 (13.5)
Sector (n=333), n (%)	
Public	298 (89.5)
Private	17 (5.1)
Public and private	18 (5.4)
Local patient blood management educational events in the previous 3 years (n=348), n (%)	
Yes	136 (39.1)
No	170 (48.9)
Unsure	42 (12.1)
Anemia diagnostic tests available (of 7; n=350), median (IQR)	5 (4‐5)

a Descriptive data were previously reported in the primary survey publication [13] and are reproduced here to characterize the analytical sample.

b Physician grade and hospital category were end-of-survey items and had more dropout than the earlier educational and diagnostic items.

### PBM Adoption

Overall, 73.7% (373/506) of respondents reported applying PBM, 33.1% (158/477) worked in a hospital with a formal PBM group, and 33.5% (117/349) reported routine preoperative anemia treatment in their hospital, reproducing the headline figures of the primary report. Unadjusted variation by hospital category, grade, and educational exposure is shown in Table 2.

**Table 2.** Adoption outcomes by hospital category and physician grade (unadjusted proportions)^a^.

Stratum	Applies PBM^b^, n/N (%)	Formal PBM group, n/N (%)	Routine preoperative anemia, n/N (%)
Hospital category			
University or institute	102/130 (78.5)	48/130 (36.9)	46/130 (35.4)
County	64/90 (71.1)	21/91 (23.1)	19/91 (20.9)
Municipal or town	44/67 (65.7)	18/67 (26.9)	20/66 (30.3)
Other	38/44 (86.4)	21/45 (46.7)	29/45 (64.4)
Physician grade			
Senior consultant	141/173 (81.5)	58/175 (33.1)	61/174 (35.1)
Specialist	78/112 (69.6)	30/112 (26.8)	37/112 (33)
Resident	27/44 (61.4)	18/44 (40.9)	15/44 (34.1)

a Descriptive data were previously reported in the primary survey publication [13] and are reproduced here to characterize the analytical sample. Univariable associations with local educational exposure are reported in Multimedia Appendix 2.

b PBM: patient blood management.

In adjusted models (Table 3 and Figure 2), local PBM educational events were the strongest and only consistently significant modifiable correlate across all 3 outcomes. Hospital category was largely not associated with the outcomes after adjustment, and residents were less likely than senior consultants to report applying PBM. Model discrimination was acceptable (AUC 0.77, 0.77, and 0.73). The models included 326 respondents (243 outcome events) for reported PBM application, 328 respondents (106 events) for a formal PBM group, and 327 respondents (113 events) for routine preoperative anemia treatment. The “other hospital” category was associated with routine preoperative anemia treatment but not with reported PBM application or a formal PBM group. This category was small and heterogeneous (n=45); 20 respondents (44.4%) worked wholly or partly in the private sector. Compared with respondents in university hospitals, those in the “other” category more often reported outpatient preanesthetic assessment (23/44, 52.3% vs 52/130, 40%) and consultation more than 7 days before surgery (29/45, 64.4% vs 54/130, 41.5%). These descriptive differences suggest possible pathway and case-mix explanations but do not establish why the association occurred.

**Table 3.** Adjusted odds ratios (aORs) from the primary multivariable logistic regression models^a^.

Predictor	Applies PBM^b^, aOR (95% CI)	*P* value	Formal PBM group, aOR (95% CI)	*P* value	Routine preoperative anemia, aOR (95% CI)	*P* value
Educational events						
Yes (reference: no)	9.78 (4.33‐22.07)	<.001	7.72 (4.30‐13.88)	<.001	3.68 (2.12‐6.39)	<.001
Unsure (reference: no)	1.84 (0.82‐4.10)	.14	1.23 (0.48‐3.14)	.67	1.38 (0.59‐3.23)	.45
Physician grade						
Specialist (reference: senior consultant)	0.73 (0.39‐1.36)	.32	1.19 (0.64‐2.21)	.58	1.31 (0.73‐2.33)	.36
Resident (reference: senior consultant)	0.32 (0.14‐0.74)	.008	2.21 (0.97‐5.07)	.06	1.39 (0.63‐3.07)	.41
Hospital category						
County (reference: university)	0.95 (0.48‐1.89)	.89	0.73 (0.37‐1.42)	.35	0.60 (0.31‐1.14)	.12
Municipal or town (reference: university)	0.65 (0.31‐1.39)	.27	0.99 (0.45‐2.18)	.98	1.07 (0.52‐2.21)	.85
Other (reference: university)	1.86 (0.67‐5.18)	.24	2.19 (0.99‐4.86)	.05	4.29 (1.99‐9.27)	<.001

a *P* values are from 2-sided Wald tests. The model for reported patient blood management (PBM) application included 326 respondents and 243 events (area under the receiver operating characteristic curve [AUC] 0.77); the model for a formal PBM group included 328 respondents and 106 events (AUC 0.77); and the model for routine preoperative anemia treatment included 327 respondents and 113 events (AUC 0.73).

b PBM: patient blood management.

**Figure 2.**
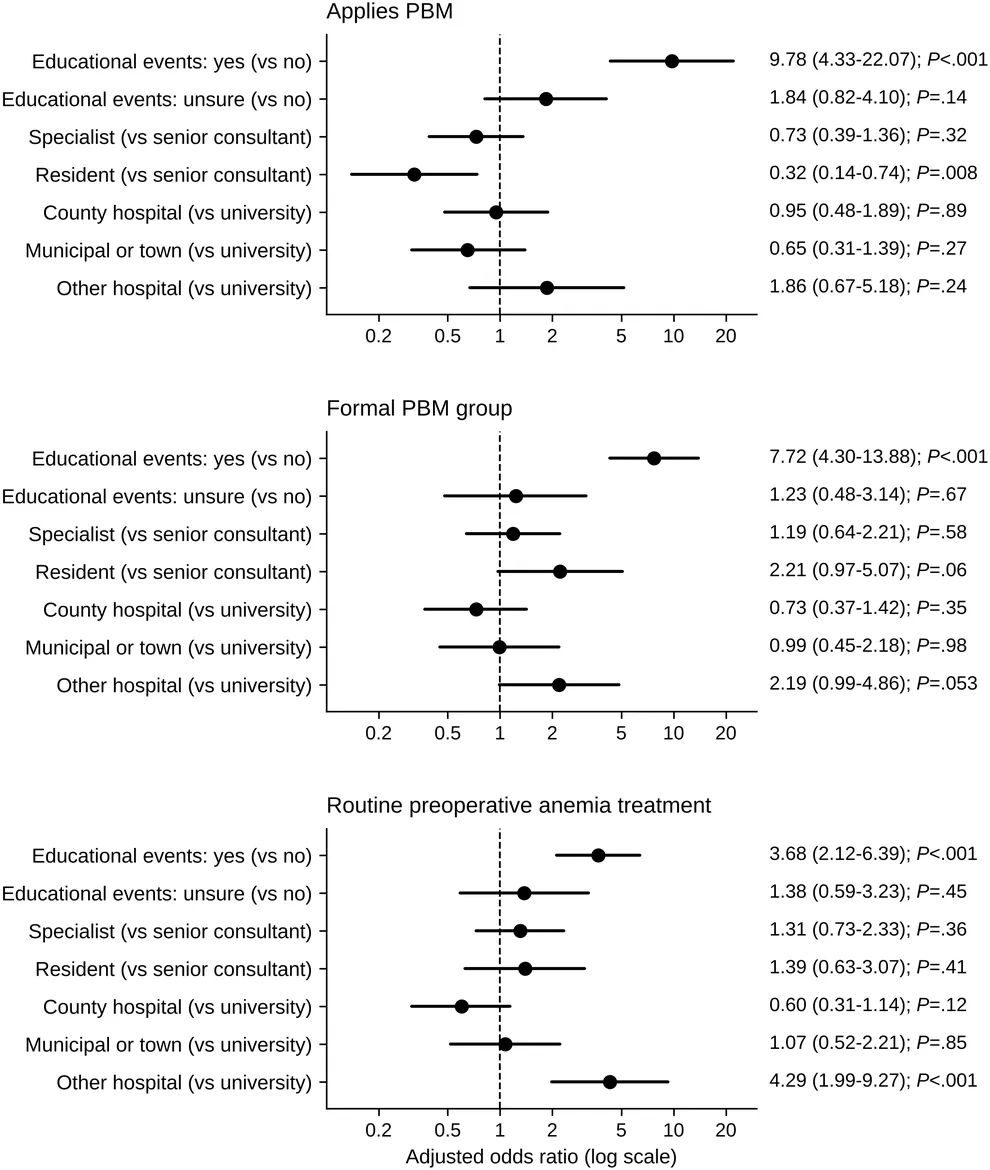
Adjusted predictors of the 3 adoption outcomes. Points are adjusted odds ratios, horizontal lines are 95% CIs, and corresponding 2-sided Wald *P* values are shown. PBM: patient blood management.

### Timing of the Preanesthetic Consultation

The reported timing of the preanesthetic consultation was itself associated with PBM adoption (n=326‐328). Respondents who more often assessed patients well before surgery were more likely to report routine preoperative anemia treatment in their hospital. Each 1-level increase in the frequency of consulting more than 7 days before surgery carried an adjusted odds ratio (aOR) of 1.79 (95% CI 1.36‐2.35) for routine preoperative anemia treatment and 1.87 (95% CI 1.33‐2.65) for applying PBM. Both models adjusted for physician grade, hospital category, and local educational events. Crudely, routine preoperative anemia treatment was reported by 54.1% (66/122) of respondents who performed the more-than-7-day consultation at least sometimes, versus 22.1% (50/226) of those who did so rarely or never. The gradient reversed for late assessment: more frequent consultation on the morning of surgery was associated with lower odds of routine preoperative anemia treatment (aOR 0.75 per level, 95% CI 0.59‐0.95). Consultation timing was not associated with the presence of a formal PBM group (aOR 1.13 per level for the more-than-7-day item, 95% CI 0.86‐1.48). The per-level associations had *P*<.001 for routine preoperative anemia treatment, *P*<.001 for applying PBM, *P*=.39 for the formal PBM group, and *P*=.02 for morning-of-surgery consultation. Treating the timing score as categorical rather than linear did not improve model fit (likelihood-ratio *P*=.82 for preoperative anemia treatment and *P*=.86 for applying PBM). Category-specific ORs also rose monotonically: for preoperative anemia treatment, they were 1.48, 3.54, and 4.95 for “rarely,” “sometimes,” and “usually” versus “never.” Both findings support the linear coding (Multimedia Appendix 2).

### Transfusion-Trigger Concordance

Concordance with the study-defined restrictive reference varied across scenarios (Table 4 and Figure 3). Two patterns were consistent. First, concordance was lower for older than younger patients in otherwise identical scenarios (active bleeding without cardiac disease: 195/345, 56.5% at ≥65 years vs 288/347, 83% at <65 years). In the scenario involving an older patient with cardiac disease and active bleeding, 42.7% (148/347) selected hemoglobin ≥9 g/dL. Second, among older patients without cardiac disease, respondents selected more liberal thresholds during active bleeding than in the corresponding scenario without active bleeding (McNemar *P*<.001). Because fixed thresholds are least directly applicable during active bleeding, this contrast describes a difference in stated numerical triggers rather than a measure of decision quality. Overall, 56.5% (196/347) endorsed a liberal trigger (hemoglobin ≥8 g/dL) in at least 1 noncardiac scenario.

A restrictive pattern was present in 203 (59.7%) of the 340 respondents who answered all 8 vignettes. In the complete-case adjusted model (n=323), self-reported PBM application was not associated with a restrictive pattern (aOR 1.17, 95% CI 0.69‐1.98; *P*=.56). Thus, reported PBM application did not correspond to more restrictive stated thresholds in these hypothetical scenarios.

**Table 4.** Per-scenario concordance, defined as agreement of the stated trigger with the study-defined restrictive hemoglobin reference.

Scenario	Concordant, n (%)
≥65 y, noncardiac disease, and active bleeding (n=345)	195 (56.5)
≥65 y, cardiac disease, and active bleeding (n=347)	199 (57.3)
<65 y, noncardiac disease, and active bleeding (n=347)	288 (83.0)
<65 y, cardiac disease, and active bleeding (n=347)	255 (73.5)
≥65 y, noncardiac disease, and no active bleeding (n=344)	229 (66.6)
≥65 y, cardiac disease, and no active bleeding (n=343)	252 (73.5)
<65 y, noncardiac disease, and no active bleeding (n=345)	289 (83.8)
<65 y, cardiac disease, and no active bleeding (n=345)	286 (82.9)

**Figure 3.**
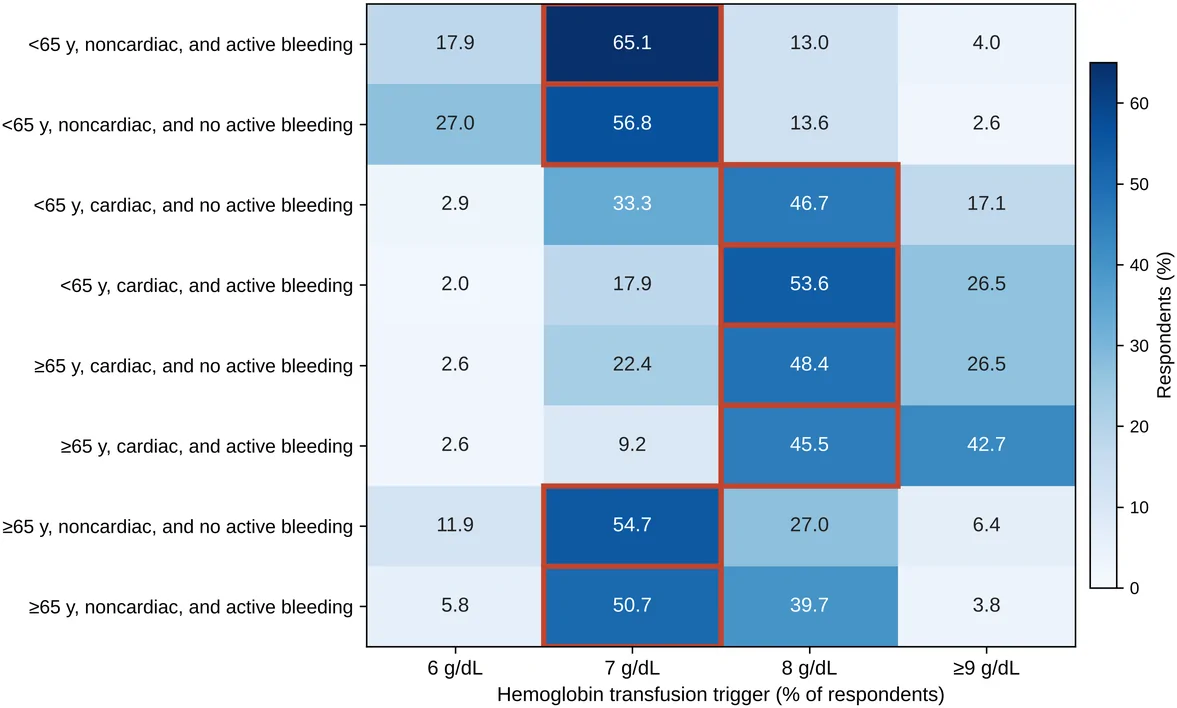
Distribution of stated hemoglobin transfusion triggers across the 8 scenarios. The outlined cell in each row marks the study-defined restrictive reference; numbers are percentages of respondents. “No active bleeding” reproduces the questionnaire wording and does not imply that hemodynamic stability or symptoms were specified.

### Sensitivity Analyses

The direction of all findings was unchanged under the alternative concordance-scoring rules (Table 5); only the absolute level of measured concordance changed, as expected. Under the strict rule, 27.6% (94/340) met the restrictive pattern definition, versus 59.7% (203/340) under the primary rule and 97.9% (333/340) under the lenient rule. The age and bleeding state gradients persisted under all 3 rules.

For the formal PBM group outcome, in which “don’t know” responses (91/477, 19.1%) were counted as absence of a group in the primary analysis, a sensitivity model excluding those responses (386 eligible; complete-case regression n=272) gave a materially similar educational events effect (aOR 8.44, 95% CI 4.55‐15.65, vs 7.72, 95% CI 4.30‐13.88 in the primary model). The only appreciable change was a strengthening of the resident association (aOR 3.56, 95% CI 1.31‐9.64, vs 2.21, 95% CI 0.97‐5.07). This is consistent with residents being more likely to answer “don’t know,” which itself indicates the limited visibility of PBM structures to trainees.

**Table 5.** Concordance under the 3 per-vignette scoring rules among the 340 respondents who answered all 8 vignettes^a^.

Scoring rule	Per-scenario concordance, mean (%; SD)	Concordant scenarios (of 8), median (IQR)	Restrictive pattern (≥6/8), n (%)
Primary (less than or equal to the reference)	72.1 (11.1)	6 (4-8)	203 (59.7)
Strict (equal to the reference)	52.9 (6.2)	4 (3-6)	94 (27.6)
Lenient (±1 g/dL)	96.8 (1.4)	8 (8-8)	333 (97.9)

a Mean per-scenario concordance is the average of the 8 scenario-level proportions; the restrictive pattern is the respondent-level classification (≥6 of 8 vignettes concordant under the rule in use). The ≥9 g/dL option was coded as 9 g/dL.

## Discussion

### Principal Findings

This secondary analysis of a national survey yields 3 findings that the descriptive primary report did not reveal. First, reported adoption of PBM in Romania was associated far more strongly with local educational activity than with hospital size or seniority. Hospitals that had held PBM educational events showed roughly 10-fold higher odds of applying PBM and 8-fold higher odds of having a formal PBM group, independent of hospital category and grade. Second, stated transfusion triggers were systematically more liberal for older patients than for younger patients in otherwise identical situations, and most respondents endorsed a liberal threshold in at least 1 noncardiac scenario. Third, and most importantly for implementation, self-reported application of PBM was not associated with a restrictive pattern of stated thresholds—reported adoption and stated bedside thresholds were disconnected.

### Comparison With Prior Work

The primary survey report [13] and international surveys [16] documented incomplete PBM uptake, while a Romanian study identified organizational barriers to implementation [17]. The present analysis extends that work by examining correlates of reported adoption and by quantifying threshold concordance. The association with educational exposure is consistent with evidence supporting locally embedded education reinforced by audit and feedback [18,19]. Preoperative anemia treatment is likewise supported by evidence linking anemia to adverse surgical outcomes and by consensus and trial data on correction strategies [20-22].

Respondents selected more liberal thresholds for older patients, although major trials and meta-analyses have generally not shown harm from restrictive strategies in older or cardiovascular populations [5,10,23]. In the prespecified older subgroup of the Transfusion Requirements in Cardiac Surgery III trial, a restrictive strategy was associated with a lower risk of the composite clinical outcome than a liberal strategy (patients aged 75 years or older: OR 0.70, 95% CI 0.54‐0.89). Respondents may nevertheless have implicitly attributed frailty or unreported comorbidity to older patients. Cardiac disease was held constant in the factorial design, but such implicit assumptions cannot be excluded in a stated-preference survey.

PBM also encompasses perioperative bleeding management, including tranexamic acid and cell salvage [24,25]. Variation in stated red blood cell thresholds is consistent with evidence that transfusion practice remains heterogeneous across institutions and specialties [26]. Registry data further suggest that transfusion need is concentrated in a minority of patients, supporting risk-stratified implementation [27]. Together, these findings indicate that closing the gap between reported adoption and stated practice requires interventions directed at transfusion decisions as well as institutional structures.

The consultation timing findings offer a plausible mechanism for the low rate of preoperative anemia treatment. Correcting anemia before surgery, whether with iron or by rescheduling, requires lead time. Respondents who routinely assessed patients more than 7 days before surgery were substantially more likely to report routine preoperative anemia treatment in their hospital, whereas those who typically saw patients only on the morning of surgery were less likely to do so. Because this first PBM pillar is time-dependent, organizational pathways that create lead time—not clinician knowledge alone—may be a prerequisite for delivering it. Bringing the preanesthetic consultation forward is only part of the solution: anesthesiologists usually meet the patient after the surgical decision has been made, so responsibility for anemia screening is best shared with the surgical team and initiated at the surgical consultation, when the indication for surgery is established. In some centers, surgeons start the diagnostic workup and, within an agreed multidisciplinary PBM protocol, prescribe iron and selective erythropoietin; 2 recent observational studies in elective arthroplasty illustrate the feasibility of such a surgeon-initiated, shared-care pathway [28,29]. These reports should be read as examples of coordinated pathway design rather than as support for routine erythropoietin in unselected surgical patients. Such a pathway is a modifiable target that complements the educational interventions our other findings support.

The higher odds of routine preoperative anemia treatment in the “other hospital” category require cautious interpretation because university centers might be expected to lead PBM practice. The association was specific to this outcome and was not observed for reported PBM application or a formal PBM group. University hospitals had the highest reported exposure to local educational events (65/129, 50.4%), and the prevalence of formal PBM groups in university hospitals (48/130, 36.9%) exceeded that in county and municipal hospitals, although it was lower than in the “other” category (21/45, 46.7%). The “other” category was small (n=45), heterogeneous, and contained most private or mixed-sector practice in the sample (20/45, 44.4%). Respondents in this category more often reported outpatient assessment and consultation more than 7 days before surgery, which may reflect an elective case mix with more time for anemia correction. Because the study was cross-sectional and case-mix information was unavailable, these observations cannot explain the association. The association should be considered hypothesis-generating rather than evidence that nonuniversity hospitals deliver better PBM overall.

### Strengths and Limitations

Strengths include a large national sample, a balanced factorial vignette design enabling within-respondent contrasts, and sensitivity analyses addressing alternative scoring rules and attrition. Several limitations apply. The self-selected convenience sample likely overrepresented clinicians interested in PBM, potentially biasing reported adoption and concordance upward. The exact number of active email addresses and the number of invitation recipients who opened the survey were unavailable; therefore, only an approximate invitation-to-response proportion could be reported. Although respondents covered 41 of 42 county-level administrative units and included different hospital categories and physician grades, population benchmarks were unavailable, so representativeness by geography, institution, and professional seniority could not be established. Reported PBM application was measured with a single item that did not define the 3 PBM pillars and may overestimate implementation of the complete bundle. Similarly, the survey measured the reported presence of formal structures, not their activity. Vignette responses reflect stated intent rather than observed transfusion practice, and concordance with a study-defined hemoglobin reference is not equivalent to the appropriateness of an individual transfusion decision, which also depends on hemodynamic status, bleeding rate, symptoms, anticipated blood loss, and comorbidity; fixed hemoglobin thresholds are least directly applicable during active bleeding, so discordance in the bleeding vignettes should not be equated with inappropriate practice. Cross-sectional associations may be bidirectional: hospitals already committed to PBM may be more likely to organize education, so the education-adoption association should not be interpreted causally. PBM is multidisciplinary, but only anesthesiologists were surveyed; conclusions about hospital-level implementation must therefore be limited to their perspective. The survey did not measure transfusion rates or clinical outcomes. Finally, the analysis was not prospectively registered and should be considered exploratory and hypothesis-generating.

### Practical Implications

These findings suggest 2 complementary implementation priorities. First, locally delivered multidisciplinary education may support reported PBM adoption, but its effectiveness should be evaluated prospectively because the observed association may reflect reverse causation. Second, implementation should address the transfusion decision through approaches such as decision support, single-unit ordering, and audit and feedback, particularly in scenarios involving older patients. Earlier preanesthetic assessment may also create the lead time required for anemia treatment, and anemia screening initiated at the surgical consultation under a shared anesthetic-surgical PBM protocol may help secure that lead time [28,29]. A coordinated national program could involve anesthesiology, surgery, hematology, transfusion medicine, hospital management, residency training, and institutional PBM champions, potentially supported by dedicated funding, for which the economic case of preoperative anemia management in the Romanian setting has been modeled [30]. Linking survey responses to audited process indicators, transfusion use, and clinical outcomes, building on earlier work characterizing perioperative hemoglobin trajectories and predictors of transfusion [31], would allow the relationship between stated and observed practice to be tested directly.

### Conclusions

Five years after publication of national recommendations, most responding Romanian anesthesiologists reported applying PBM, while only approximately one-third reported a formal institutional PBM group or routine preoperative anemia treatment. Local educational activity and earlier preanesthetic assessment were associated with reported implementation outcomes, but the cross-sectional design precludes causal interpretation. Self-reported PBM application was not associated with concordance with the study-defined restrictive reference in hypothetical scenarios, particularly those involving older patients. Sharing responsibility for early anemia screening with the surgical team is a practical route to the lead time that anemia correction requires. Prospective multidisciplinary studies using hospital-level process indicators, observed transfusion practice, and clinical outcomes are required.

## Supplementary material

10.2196/106731 Multimedia Appendix 1 Patient blood management survey Romania—raw questions.

10.2196/106731 Multimedia Appendix 2 Full model outputs supporting the analyses reported in the main text.

## Abbreviations

aOR: adjusted odds ratio
AUC: area under the receiver operating characteristic curve
CHERRIES: Checklist for Reporting Results of Internet E-Surveys
OR: odds ratio
PBM: patient blood management
SRATI: Romanian Society of Anaesthesia and Intensive Care
