# Supplementary material for: Patient Blood Management Adoption and Transfusion-Threshold Concordance Among Romanian Anesthesiologists: National Survey Analysis
Source: JMIR Perioper Med. 2026 Oct 16;9:e106731. doi: 10.2196/106731 (PMC13674520; doi:10.2196/106731)
Supplement: Supplementary file 1 [file periop-v9-e106731-s001.pdf]

Multimedia Appendix 2

Survey items used in this secondary analysis: Romanian original and English translation

The full questionnaire comprised 38 items (37 closed-response items and 1 open-ended item); the primary report describes the complete instrument. Reproduced below are the items analysed in this secondary analysis, in the original Romanian with English translations, grouped by construct. Item numbers correspond to those used in the manuscript. In item Q7 the "rarely" option was worded "Rar" and in items Q8–Q10 and Q13 "Rareori"; both mean "rarely" and were coded identically.

A. PBM adoption and institutional context

Item	Romanian (original)	English translation
Q2	Aplicați PBM în practica dumneavoastră curentă?<br>*Options: Da / Nu*	Do you apply PBM in your current practice?<br>*Options: Yes / No*
Q4	În spitalul dumneavoastră, există un grup de implementare al PBM oficial?<br>*Options: Da / Nu / Nu știu*	Is there an official PBM implementation group in your hospital?<br>*Options: Yes / No / I don't know*
Q6	În spitalul dumneavostră s-au organizat evenimente educaționale despre PBM în ultimii 3 ani?<br>*Options: Da / Nu / Nu știu*	Have educational events on PBM been organised in your hospital in the last 3 years?<br>*Options: Yes / No / I don't know*

B. Pre-anaesthetic assessment and preoperative anaemia management

Item	Romanian (original)	English translation
Q7	În practica dumneavoastră curentă, efectuați consultația pre-anestezică cu mai mult de 7 zile înainte de operație?<br>*Options: Niciodată / Rar / Câteodată / De obicei / Întotdeauna*	In your current practice, do you perform the pre-anaesthetic consultation more than 7 days before surgery?<br>*Options: Never / Rarely / Sometimes / Usually / Always*
Q8	În practica dumneavoastră curentă, efectuați consultația pre-anestezică cu mai mult de o zi, dar mai puțin de 7 zile înainte de operație?<br>*Options: Niciodată / Rareori / Câteodată / De obicei / Întotdeauna*	In your current practice, do you perform the pre-anaesthetic consultation more than one day but less than 7 days before surgery?<br>*Options: Never / Rarely / Sometimes / Usually / Always*
Q9	În practica dumneavoastră curentă, efectuați consultația pre-anestezică în pre-ziua operației?<br>*Options: Niciodată / Rareori / Câteodată / De obicei / Întotdeauna*	In your current practice, do you perform the pre-anaesthetic consultation on the day before surgery?<br>*Options: Never / Rarely / Sometimes / Usually / Always*
Q10	În practica dumneavoastră curentă, efectuați consultația pre-anestezică în dimineața operației?<br>*Options: Niciodată / Rareori / Câteodată / De obicei / Întotdeauna*	In your current practice, do you perform the pre-anaesthetic consultation on the morning of surgery?<br>*Options: Never / Rarely / Sometimes / Usually / Always*
Q11	În spitalul dumneavoastră, există posibilitatea evaluării pre-anestezice în regim ambulator?<br>*Options: Da / Nu*	Does your hospital offer the possibility of outpatient pre-anaesthetic assessment?<br>*Options: Yes / No*
Q12	În evaluarea pre-operatorie a anemiei, care din următoarele teste vă sunt accesibile în instituția dumneavoastră (mai multe variante pot fi selectate)?<br>*Options: Hemograma; Sideremie; Feritina; Coeficientul de saturație a transferinei; Receptorul solubil de transferină; Numărătoare de reticulocite;*	In the preoperative evaluation of anaemia, which of the following tests are accessible in your institution (multiple options may be selected)?<br>*Options: Full blood count; Serum iron; Ferritin; Transferrin saturation; Soluble transferrin receptor; Reticulocyte count; C-reactive protein*

	Proteina C reactivă	
Q13	În spitalul dumneavoastră, se practică tratamentul pre-operator al anemiei (altul decât transfuzia de sânge)?<br>*Options: Niciodată / Rareori / Câteodată / De obicei / Întotdeauna*	In your hospital, is preoperative treatment of anaemia (other than blood transfusion) practised?<br>*Options: Never / Rarely / Sometimes / Usually / Always*

C. Transfusion-trigger clinical vignettes (2 × 2 × 2 factorial: age × cardiac disease × bleeding state)

Response options for all eight vignettes: 6 g/dL / 7 g/dL / 8 g/dL / 9 g/dL or higher (Romanian: "6 g/dl", "7 g/dl", "8 g/dl", "9 g/dl sau mai mare").

Item	Romanian (original)	English translation
Q22	În cazul sângerării intra-operatorii, la un pacient în vârstă de 65 ani sau mai mult, fără patologie cardiacă, la ce valoare a hemoglobinei transfuzați de rutină?<br>*Options: 6 g/dl / 7 g/dl / 8 g/dl / 9 g/dl sau mai mare*	In the case of intraoperative bleeding, in a patient aged 65 years or older, without cardiac disease, at what haemoglobin value do you routinely transfuse?<br>*Options: 6 g/dL / 7 g/dL / 8 g/dL / 9 g/dL or higher*
Q23	În cazul sângerării intra-operatorii, la un pacient în vârstă de 65 ani sau mai mult, cu patologie cardiacă cunoscută, la ce valoare a hemoglobinei transfuzați de rutină?<br>*Options: ca Q22*	In the case of intraoperative bleeding, in a patient aged 65 years or older, with known cardiac disease, at what haemoglobin value do you routinely transfuse?<br>*Options: as Q22*
Q24	În cazul sângerării intra-operatorii, la un pacient mai tânăr de 65 ani, fără patologie cardiacă, la ce valoare a hemoglobinei transfuzați de rutină?<br>*Options: ca Q22*	In the case of intraoperative bleeding, in a patient younger than 65 years, without cardiac disease, at what haemoglobin value do you routinely transfuse?<br>*Options: as Q22*
Q25	În cazul sângerării intra-operatorii, la un pacient mai tânăr de 65 ani, cu patologie cardiacă cunoscută, la ce valoare a hemoglobinei transfuzați de rutină?<br>*Options: ca Q22*	In the case of intraoperative bleeding, in a patient younger than 65 years, with known cardiac disease, at what haemoglobin value do you routinely transfuse?<br>*Options: as Q22*
Q26	În cazul unui pacient de peste 65 ani, fără patologie cardiacă, anemic, fără sângerare activă, care este valoarea Hb la care transfuzați de rutină?<br>*Options: ca Q22*	In the case of a patient over 65 years, without cardiac disease, anaemic, without active bleeding, what is the Hb value at which you routinely transfuse?<br>*Options: as Q22*
Q27	În cazul unui pacient de peste 65 ani, cu patologie cardiacă cunoscută, anemic, fără sângerare activă, care este valoarea Hb la care transfuzați de rutină?<br>*Options: ca Q22*	In the case of a patient over 65 years, with known cardiac disease, anaemic, without active bleeding, what is the Hb value at which you routinely transfuse?<br>*Options: as Q22*
Q28	În cazul unui pacient mai tânăr de 65 ani, fără patologie cardiacă, anemic, fără sângerare activă, care este valoarea Hb la care transfuzați de rutină?<br>*Options: ca Q22*	In the case of a patient younger than 65 years, without cardiac disease, anaemic, without active bleeding, what is the Hb value at which you routinely transfuse?<br>*Options: as Q22*
Q29	În cazul unui pacient mai tânăr de 65 ani, cu patologie cardiacă cunoscută, anemic, fără sângerare activă, care este valoarea Hb la care transfuzați de rutină?<br>*Options: ca Q22*	In the case of a patient younger than 65 years, with known cardiac disease, anaemic, without active bleeding, what is the Hb value at which you routinely transfuse?<br>*Options: as Q22*

D. Demographic and institutional items (demographic block at the end of the questionnaire; referred to in the manuscript by construct, not by item number)

Item	Romanian (original)	English translation
D1	În ce fel de spital lucrați:<br>*Options: Institut/ Spital Universitar / Spital Județean / Spital Municipal/Orășenesc / Altul*	In what kind of hospital do you work?<br>*Options: Institute/University hospital / County hospital / Municipal/Town hospital / Other*
D2	Practica dumneavoastră se desfășoară într-un spital:<br>*Options: Public / Privat / Ambele*	Your practice takes place in a hospital that is:<br>*Options: Public / Private / Both*
D3	Sunteți medic:<br>*Options: Primar / Specialist / Rezident*	You are a physician with the grade of:<br>*Options: Senior consultant (primar) / Specialist / Resident*
D4	In ce judet lucrati?<br>*Options: listă de județe*	In which county do you work?<br>*Options: list of counties*
