# Supplementary material for: Patient Blood Management Adoption and Transfusion-Threshold Concordance Among Romanian Anesthesiologists: National Survey Analysis
Source: JMIR Perioper Med. 2026 Oct 16;9:e106731. doi: 10.2196/106731 (PMC13674520; doi:10.2196/106731)
Supplement: Supplementary file 2 [file periop-v9-e106731-s002.pdf]

Multimedia Appendix 1

Supplementary regression tables

Full model outputs supporting the analyses reported in the main text. Odds ratios (OR) and rate ratios are shown with 95% confidence intervals and exact P values (Wald tests), reported to 2 decimal places, to 3 decimal places when below .01 or between .045 and .055, and as P<.001 when below .001.

Table S1. Univariable (unadjusted) associations with the three adoption outcomes

Predictor (unadjusted OR, 95% CI; P)	Applies PBM	Formal group	Routine preop anaemia
Educational events: Yes (ref: No)	8.32 (3.95–17.52); P<.001	6.35 (3.76–10.72); P<.001	3.37 (2.06–5.53); P<.001
Educational events: unsure	1.19 (0.58–2.43); P=.64	1.14 (0.48–2.72); P=.76	1.21 (0.56–2.64); P=.62
Specialist (ref: senior consultant)	0.52 (0.30–0.91); P=.02	0.74 (0.44–1.25); P=.26	0.91 (0.55–1.51); P=.72
Resident (ref: senior consultant)	0.36 (0.18–0.74); P=.005	1.40 (0.71–2.75); P=.34	0.96 (0.48–1.92); P=.90
County (ref: University)	0.68 (0.36–1.25); P=.21	0.51 (0.28–0.94); P=.03	0.48 (0.26–0.90); P=.02
Municipal/town (ref: University)	0.53 (0.27–1.01); P=.054	0.63 (0.33–1.20); P=.16	0.79 (0.42–1.50); P=.48
Other (ref: University)	1.74 (0.67–4.53); P=.26	1.49 (0.75–2.97); P=.25	3.31 (1.63–6.72); P<.001
Private (ref: Public)	5.32 (0.69–40.93); P=.11	3.25 (1.20–8.81); P=.02	3.92 (1.41–10.91); P=.009
Public+private (ref: Public)	1.15 (0.36–3.64); P=.81	1.45 (0.54–3.85); P=.46	1.90 (0.71–5.08); P=.20
Outpatient pre-anaesthesia assessment	1.89 (1.07–3.34); P=.03	2.47 (1.54–3.96); P<.001	3.40 (2.11–5.49); P<.001
Anaemia-diagnostic tests (per test)	1.25 (1.05–1.48); P=.01	1.24 (1.05–1.47); P=.01	1.31 (1.10–1.55); P=.002

Table S1. Each row is from a separate univariable logistic regression. Categorical predictors are shown relative to their reference level; continuous predictors (outpatient assessment as 0/1; diagnostic-test count) are per unit. Hospital sector (private; public+private) is shown for completeness as univariable associations only; it was not entered into any multivariable model because it was collinear with hospital category (private practice occurred almost exclusively within the "other" category).

Table S2. Extended multivariable models (core covariates plus infrastructure indices)

Predictor (adjusted OR, 95% CI; P)	Applies PBM	Formal group	Routine preop anaemia
Educational events: Yes (ref: No)	8.92 (3.92–20.31); P<.001	7.08 (3.91–12.82); P<.001	3.17 (1.80–5.58); P<.001
Educational events: unsure	1.79 (0.80–4.01); P=.16	1.19 (0.46–3.05); P=.72	1.30 (0.55–3.09); P=.55
Specialist (ref: senior consultant)	0.71 (0.38–1.33); P=.29	1.18 (0.63–2.20); P=.60	1.30 (0.72–2.36); P=.38
Resident (ref: senior consultant)	0.29 (0.12–0.69); P=.005	2.01 (0.86–4.69); P=.11	1.22 (0.54–2.78); P=.63
County (ref: University)	1.05 (0.52–2.10); P=.90	0.80 (0.40–1.57); P=.51	0.70 (0.36–1.36); P=.29
Municipal/town (ref: University)	0.72 (0.33–1.56); P=.41	1.10 (0.49–2.47); P=.81	1.35 (0.63–2.87); P=.43
Other (ref: University)	1.70 (0.60–4.81); P=.32	1.89 (0.84–4.28); P=.12	3.63 (1.64–8.03); P=.002
Outpatient pre-anaesthesia assessment	1.59 (0.81–3.13); P=.18	1.75 (1.00–3.08); P=.051	2.49 (1.45–4.28); P<.001
Anaemia-diagnostic tests (per test)	1.08 (0.88–1.33); P=.44	1.06 (0.87–1.29); P=.58	1.18 (0.97–1.43); P=.10

Table S2. Multivariable logistic regression adjusting for grade, hospital category, educational events, outpatient pre-anaesthesia assessment and the anaemia-diagnostic-test count (hospital sector was not entered, being collinear with hospital category). Model n: applies 325, formal group 327, preop anaemia 326; AUC 0.77, 0.77, 0.75 respectively. The educational-events association is unchanged from the core model, confirming it is not explained by institutional infrastructure.

Table S3. Formal-PBM-group outcome: primary model vs sensitivity model excluding "don't know"

Predictor (adjusted OR, 95% CI; P)	Primary (don't know = no)	Sensitivity (don't know excluded)
Educational events: Yes (ref: No)	7.72 (4.30–13.88); P<.001	8.44 (4.55–15.65); P<.001
Educational events: unsure	1.23 (0.48–3.14); P=.67	2.38 (0.84–6.76); P=.10
Specialist (ref: senior consultant)	1.19 (0.64–2.21); P=.58	1.20 (0.62–2.33); P=.59
Resident (ref: senior consultant)	2.21 (0.97–5.07); P=.06	3.56 (1.31–9.64); P=.01

County (ref: University)	0.73 (0.37–1.42); P=.35	0.69 (0.33–1.44); P=.33
Municipal/town (ref: University)	0.99 (0.45–2.18); P=.98	0.75 (0.33–1.70); P=.49
Other (ref: University)	2.19 (0.99–4.86); P=.053	1.67 (0.72–3.84); P=.23

Table S3. Primary model n=328 (106 events); sensitivity model complete-case n=272 (106 events; 386 respondents were eligible after excluding "don't know" answers, of whom 272 had complete covariate data). The educational-events effect is materially unchanged; the resident association strengthens when "don't know" responses are excluded, consistent with residents being more likely to report not knowing whether a formal group exists.

Table S4. Predictors of transfusion-trigger behaviour

S4a. Restrictive pattern (≥6/8 vignettes concordant) — logistic regression

Predictor	Adjusted OR (95% CI); P
Applies PBM (yes vs no)	1.17 (0.69–1.98); P=.56
Specialist (ref: senior consultant)	0.90 (0.54–1.49); P=.68
Resident (ref: senior consultant)	0.68 (0.34–1.38); P=.29
County (ref: University)	0.86 (0.49–1.51); P=.60
Municipal/town (ref: University)	0.79 (0.41–1.52); P=.47
Other (ref: University)	0.77 (0.38–1.58); P=.48

S4b. Number of concordant scenarios (0–8) — quasi-Poisson regression

Predictor	Rate ratio (95% CI); P
Educational events: Yes (ref: No)	0.96 (0.87–1.05); P=.37
Educational events: unsure	0.82 (0.71–0.95); P=.009
Specialist (ref: senior consultant)	1.00 (0.91–1.10); P=.94
Resident (ref: senior consultant)	0.98 (0.85–1.12); P=.72
County (ref: University)	0.96 (0.87–1.07); P=.50
Municipal/town (ref: University)	0.94 (0.83–1.07); P=.35
Other (ref: University)	0.94 (0.82–1.07); P=.33

Table S4. S4a models the restrictive pattern among respondents answering all eight vignettes (n=323 with complete covariates). S4b models the count of concordant scenarios by quasi-Poisson regression (n=321), accounting for over- or under-dispersion.

Table S5. Consultation-timing predictors of PBM adoption (multivariable models)

Predictor (adjusted OR, 95% CI; P)	Applies PBM	Formal group	Routine preop anaemia
Consultation >7 days before surgery, per level	1.87 (1.33–2.65); P<.001	1.13 (0.86–1.48); P=.39	1.79 (1.36–2.35); P<.001
Consultation >7 days before surgery, at least sometimes vs rarely/never	2.61 (1.28–5.30); P=.008	1.22 (0.69–2.16); P=.49	3.16 (1.84–5.43); P<.001
Consultation on the morning of surgery, per level	0.81 (0.63–1.04); P=.10	0.97 (0.76–1.23); P=.80	0.75 (0.59–0.95); P=.02

Table S5. Each cell is the adjusted odds ratio for the stated consultation-timing predictor, from a separate multivariable logistic regression additionally adjusting for physician grade, hospital category and local PBM educational events. Timing items were scored never=0, rarely=1, sometimes=2, usually=3, always=4; "per level" gives the OR for a one-category increase. Applies-PBM model n=326; formal-group n=328; routine-preoperative-anaemia n=327.

Table S6. Linearity check for the ordinal consultation-timing predictor (Q7, >7 days before surgery)

Outcome	Rarely vs never	Sometimes vs never	Usually vs never	Always vs never	Linear (per level)	LR test linear vs categorical
Routine preoperative anaemia treatment	1.48 (0.74–2.96)	3.54 (1.71–7.33)	4.95 (1.90–12.89)	6.87 (0.65–72.60)	1.79 (1.36–2.35); P<.001	χ^2=0.94, P=.82
Applies PBM	2.21 (1.14–4.27)	3.20 (1.41–7.28)	6.13 (1.27–29.63)	not estimable (separation)	1.87 (1.33–2.65); P<.001	χ^2=0.78, P=.86

Table S6. Category-specific adjusted odds ratios (versus "never") from models in which the Q7 frequency score entered as a categorical variable, compared with the linear (per-level) coding; all models adjust for grade, hospital category and educational events. The likelihood-ratio test compares the categorical with the linear model (3 df). "Always" estimates are unstable because only 6 respondents chose this option (for applying PBM, all applied PBM, producing separation); the monotonic rise across informative categories and the non-significant likelihood-ratio tests support the linear coding.
